# A Heckman selection model for the safety analysis of signalized intersections

**DOI:** 10.1371/journal.pone.0181544

**Published:** 2017-07-21

**Authors:** Xuecai Xu, S. C. Wong, Feng Zhu, Xin Pei, Helai Huang, Youjun Liu

**Affiliations:** 1 School of Civil Engineering and Mechanics, Huazhong University of Science and Technology, Wuhan, China; 2 School of Civil and Environmental Engineering, Nanyang Technological University, Singapore, Singapore; 3 Department of Civil Engineering, The University of Hong Kong, Hong Kong, China; 4 Department of Automation, Tsinghua University, Beijing, China; 5 Urban Transport Research Center, School of Traffic and Transportation Engineering, Central South University, Changsha, Hunan, China; Beihang University, CHINA

## Abstract

**Purpose:**

The objective of this paper is to provide a new method for estimating crash rate and severity simultaneously.

**Methods:**

This study explores a Heckman selection model of the crash rate and severity simultaneously at different levels and a two-step procedure is used to investigate the crash rate and severity levels. The first step uses a probit regression model to determine the sample selection process, and the second step develops a multiple regression model to simultaneously evaluate the crash rate and severity for slight injury/kill or serious injury (KSI), respectively. The model uses 555 observations from 262 signalized intersections in the Hong Kong metropolitan area, integrated with information on the traffic flow, geometric road design, road environment, traffic control and any crashes that occurred during two years.

**Results:**

The results of the proposed two-step Heckman selection model illustrate the necessity of different crash rates for different crash severity levels.

**Conclusions:**

A comparison with the existing approaches suggests that the Heckman selection model offers an efficient and convenient alternative method for evaluating the safety performance at signalized intersections.

## Introduction

The simultaneous estimation of crash frequency and severity at signalized intersections is a big challenge for safety performance, which has drawn significant attention in past decades. A variety of different approaches and perspectives (Park & Lord (2007), Ye et al. (2009), Venkataraman et al. (2013)) [1–3] have been used in prediction modeling. Studies have indicated that either separate crash frequency or separate crash severity levels at signalized intersections might have led to bias in results estimations (Lord & Mannering (2010)) [4], as possible correlations between crash frequency and severity levels were not taken into account.

In general, the simultaneous estimation of crash frequency and severity includes the following methods: multi-level hierarchical structures (Kim et al. (2007)) [5], simultaneous equations (Ye et al. (2009), Kim & Washington (2006), Ye et al. (2013) [2, 6, 7] and two-stage bivariate/multivariate analysis (Park & Lord (2007), Ma & Kockelman (2006), Xu et al. (2014) [1, 8, 9], all of which can be considered either combined crash frequency/severity models, or two-stage models.

In terms of combined crash frequency/severity models, Abdel-Aty and Keller (2005) [10] initially explored crash type and severity levels simultaneously. An ordered logistic model for overall crash severity levels and a hierarchical tree-based regression model for specific crash severity levels were developed. The results indicated that the aggregation of crash types was a less effective method than the development of separate models for each level of collision. However, the results exhibited a weakness, as the two models presented were kept relatively separate with no interaction permitted. Combining the two models, Pei et al. (2011) [11] later extended and constructed a joint-probability model to integrate crash occurrence and severity predictions within a single framework. The Markov-chain Monte Carlo approach was adopted to establish a fully Bayesian estimate of the effects of the explanatory factors. The results indicated that the proposed model was appropriate for signalized intersections and roadway safety, but only the binary approach to crash severity was provided as an illustrative example. Meanwhile, the integrated models of crash frequency and severity revealed the potentiality. El-Basyouny and Sayed (2011) [12] used a multivariate Poisson-lognormal intervention model to analyze crash counts by severity levels, and extended the model to incorporate random parameters to account for the correlation between sites (Barua et al., 2016) [13]. To deal with the number of zero counts involved, Dong et al. (2014) [14] extended the multivariate zero-inflated Poisson-lognormal regression model as an alternative for modeling multivariate crash count data by severity at signalized intersections, which showed the potential of accommodating excess zeros in correlated count data. Chiou and Fu (2013) [15] addressed crash frequency and severity simultaneously in an integrated model with a multinomial generalized Poisson structure. The proposed covariance structure was shown to enhance the model’s performance. Successively Chiou and Fu (2015) [16] extended their study to address spatio-temporal dependence, which is complicated and difficult to realize.

Various studies then turned to two-stage bivariate/multivariate analysis. Wang et al. (2011) [17] adopted the less-common two-stage model to model crash frequency at different severity levels. A two-stage mixed multivariate model was proposed, and the results showed how disaggregated data at the level of individual accidents could be used to predict a certain type of low-frequency accident. Hanley and Sikka (2012) [18] explored the impact self-reporting driver distraction on the likelihood estimates of the injury severity category of crashes using a two-step correction technique, and the findings showed that self-reporting bias understates the true effect of driver distraction on injury severity. A recent study by Bhat et al. (2014) [19] integrated a count outcome model with a multinomial probit selection model, which accommodated unobserved heterogeneity and endogeneity issues at intersections. Their model can be used for crash analysis at intersections. Xu et al. (2014) [9] developed a two-stage bivariate logistic-Tobit model of crash severity and risk at different severity levels, and their results verified that the proposed model provided a good statistical fit and offered an effective alternative method for evaluating safety performance at signalized intersections. From the perspective of incident clearance time, Ding et al. (2015) [20] constructed the joint two-stage model with binary probit model and switching regression model tackling incident response time and clearance time, respectively. The results suggested that the casual effect of response time on incident clearance time would be overestimated without considering the self-selection bias. All of these studies verified that two-stage analysis provides potential for future study.

During a certain observation period, no crashes may occur at each roadway segment and intersection. Under this condition, the crash or crash rate can be considered as either zero—inflated data or censored data, i.e., left-censored at zero. As for the former, studies by Miaou (1994), Shankar et al., (1997), Carson and Mannering (2001), Lee and Mannering (2002), Lord et al. (2005, 2007) [21–26] provided and discussed the preponderance of “excess” zeros frequently observed with zero-inflated count models;Malyshkina and Mannering (2010) [27] proposed a two-state Markov switching count-data model as an alternative to deal with the preponderence of zeros and allowed for the direct estimation of the zero-crash or normal-count state in roadway segment. The results showed that Markov switching model is a viable alternative and superior to the zero-inflated models. All of models mentioned above divides the crash data into zero state and non-zero state automatically instead of by model self-selection process; as for the latter, censored data conform to the requirements of the Tobit model and has been used by scholars to conduct crash analyses. Anastasopoulos et al. (2008) [28] used the Tobit regression to address the censoring problem, and investigated the significant influencing factors of accident rates on interstate highways. Later, Anastasopoulos et al. (2012) [29] established a multivariate Tobit regression model to investigate accident-injury severity rates, and the results indicated that the multivariate Tobit model helped to analyze the factors that determined accident-injury severity on roadway segments. However, censored data, whether left- or right-censored, ignore some parts of the sample, which may not completely reflect the actual features of crashes and probably lead to erroneous estimation results.

To take advantage of the merits of two-stage analysis and address the zero-sample issue simultaneously, this study adopts a sample selection model known as the Heckman selection model. This model was presented by Heckman (1979) [30], who won the Nobel Prize for the contribution. Although various studies in the economics field have adopted this model, not so many have done so in the transportation field. Mannering and Hensher (1987) [31] provided a general overview of the discrete/continuous modeling and the application to transport analysis, which was initially used in transportation and improved our understanding of transport phenomena. Recently, Mannering and Bhat (2014) [32] summarized the evolution of methodological application and traditional data in accident research, and future methodological developments and emerging data were discussed. The issues related to unobserved heterogeneity and selectivity bias/endogeneity expand our understanding of injury severity and highway crashes. Specifically, Mannering et al. (2016) [33] focused on unobserved heterogeneity issue with various models and presented their strengths and weaknesses. However, the clearly-named study by Kaplan et al. (2016) [34] used the Heckman selection model in Denmark to explore the distance young adolescents covered by walking and cycling. Their results showed that walking and cycling necessitated different urban environments and should be encouraged for urban form planning. Based on these studies, Heckman selection model not only handles discrete/continuous modeling issue, but also addresses unobserved heterogeneity and selectivity bias/endogeneity problems simultaneously, which helps to compare the suitability of models.

The purpose of this paper is to explore a version of the Heckman selection model capable of addressing crash rate and severity at different levels simultaneously. The model provides a two-step analysis and deals with the zero-sample issue, based on which it can accommodate the heterogeneity (i.e., shared unobserved factors) between signalized intersections and then address the endogeneity (between crash rate and severity) at signalized intersections. An illustrative example using a crash dataset from signalized intersections in Hong Kong is used to evaluate the suitability of the proposed model.

## Data description

As this study aimed to investigate crash rate and severity at different levels, the crash rate and severity variables for modeling purposes were integrated into one dataset. This study used Hong Kong traffic crash data from 262 signalized intersections in two years 2002 and 2003, which were obtained from the Traffic Accident Database System (TRADS) maintained by the Hong Kong Transport Department and Hong Kong Police Force. In the analyses, the crash information of an intersection in a particular year is considered as an independent observation. There are a total of 555 observations distributed across three major districts including Hong Kong Island, Kowloon and the New Territories (see Xu et al. (2014) [9]). The counted data included intersections without crashes and one or multiple crashes with different severity levels. Specifically, if there was zero crash occurred within the signalized intersection, there was no severity level, thus only one state was recorded, i.e. one observation was counted; if there was one crash occurred, there existed at least two severity levels, one for slight injury, and the other for KSI, i.e. two observations were counted; if there was more than one crash happened, it should be determined how many crashes belonged to slight injury or KSI, and then decided how many observations were counted. Among the 555 observations, 134 exhibited zero crashes, accounting for 24% of the sample, and 421 exhibited one or more crashes, accounting for 76% of the sample.

TRADS classifies crashes as slight injury, serious and fatal. As there are few fatal crashes and both serious and fatal crashes lead to very serious injury, crashes resulting in death and serious injury are considered to fall into a single category: kill or serious injury (KSI). Among the 555 observations, 335 belonged to the slight injury category, amounting to 60% of the dataset, and 85 belonged to the KSI category, accounting for 15% of the dataset. Table 1 gives the mean and standard deviation of the crash rate for both slight injury and KSI. The crash rates for slight injury and KSI are respectively defined as the numbers of slight-injury and KSI million crashes per year divided by the annual exposure. The annual exposure is calculated by multiplying the annual average daily traffic (AADT) by 365.

**Table 1:** Sample characteristics for the selected signalized intersections.

Variable	Description	Categories			
Dependent variables					
Slight	Slight injury	Yes: 60%	No: 40%		
KSI	Killed and severe injury	Yes: 15%	No: 85%		
		Mean	Std. dev.	Min.	Max.
SCrRt	Crash rate of slight injury	0.62	0.50		
KCrRt	Crash rate of KSI	0.60	0.47		
Exposure					
AADT	AADT	35,934.16	23,219.35	903	121,221
Roadway characteristics					
Nolanes	Number of approach lanes	8.49	3.52	2	18
Noconflict	Number of conflict points	8.74	8.53	0	30
Notrnstream	Number of turning movements required	6.32	2.70	1	12
Lanewidth	Average lane width (m)	3.31	0.31	2.7	5.5
Reciprad	Reciprocal of the turning radius	0.09	0.03	0	0.2
Traffic characteristics					
Comveh	Proportion of commercial vehicles	0.21	0.10	0.01	0.66
Speed	Speed limit (km/h)	50.04	0.85	50	70
Signal-phasing scheme					
Nostages	Number of signal stages	3.14	0.78	2	7
Cycletime	Cycle time (s)	98.31	18.30	44	140
Pedcrossing	Number of pedestrian crossings	4.06	2.21	0	8
Indicator variables					
Geometrical characteristics					
2 Appr. Two approaches (Yes = 1, No = 0)		0.16		0	1
3 Appr. Three approaches (Yes = 1, No = 0)		0.30		0	1
4 Appr. Four or more approaches (Yes = 1, No = 0)		0.69		0	1
Tramstop	Presence of tram stops (Yes = 1, No = 0)	0.06		0	1
Lrtstop	Presence of LRT stops (Yes = 1, No = 0)	0.02		0	1
Road environment					
HKI	Hong Kong Island (Yes = 1, No = 0)	0.23		0	1
KLN	Kowloon (Yes = 1, No = 0)	0.58		0	1
Signal-phasing scheme					
Turningpock	Presence of a turning pocket (Yes = 1, No = 0)	0.08		0	1

Traffic volume significantly influences crash occurrence, which has been demonstrated by the non-linear relationship between crash occurrence and exposure. The AADT is therefore quantified and is expected to reveal the proportionality of the relationship between the crash rate and traffic volume.

Other influencing factors, such as geometric road design, traffic characteristics, roadway environment and signal phasing, are collected from traffic impact assessment reports made in Hong Kong. As these reports are documented for planning and design purposes, the sampling process of this study should not have been affected by any marked bias. Therefore, variables including roadway characteristics (number of approach lanes, number of conflict points, number of turning movements required, average lane width and reciprocal of the turning radius), traffic characteristics (proportion of commercial vehicles and speed limit), signal-phasing scheme (number of signal phases, signal cycle time and number of pedestrian crossings), geometric characteristics (number of approaches, presence of tram stops and light rail transit [LRT] stops), road environments on Hong Kong Island and in Kowloon and presence of turning pockets were considered. Xu et al. (2014) [9] provided a detailed description of these variables (Listed in S1 File). The available characteristics of the data sample are presented in Table 1.

## Method

The Heckman selection model is a two-equation model. First, there is the **regression model**

$$y_{i}=X_{i}\beta +\mu_{1}$$ (1)

Second, there is the **selection model**

$$Z_{i}\gamma +\mu_{2}>0$$ (2)

with the following holds:

$$\mu_{1}\sim N(0,\sigma )$$ (3a)

$$\mu_{2}\sim N(0,1)$$ (3b)

$$corr(\mu_{1},\mu_{2})=\rho$$ (3c)

where *y*_*i*_ denotes the dependent variables, *X*_*i*_ denotes the observable features of the independent variables, *β* denotes the parameters to be estimated and *μ*_1_ is a normally distributed error term with a mean of zero and a standard deviation *σ* to be estimated. *Z*_*i*_ denotes observable features including the overlapping variables with *X*_*i*_, and *γ* denotes the vectors of parameters to be estimated. *μ*_2_ is a distributed error term with a mean of zero and a standard deviation equal to one. *ρ* represents the correlation between the two error terms to be estimated. Using these two equations, samples larger than zero can be selected and estimated based on various modeling methods, through which the Heckman selection model provides consistent, asymptotically efficient estimates for all of the parameters.

In the main equation of this study, it is assumed that a **regression model** can be used to explain the crash rate for slight injury/KSI:

$$y_{i}=X_{1i}\beta_{1}+C_{i}\beta_{2}+\mu_{i}=X_{i}\beta +\mu_{i}$$ (4)

where *y*_*i*_ denotes the crash rate for slight injury/KSI; *X*_*i*_ is a vector of observable features related to slight injury/KSI, in which *X*_1*i*_ represents the endogenous variables; *C*_*i*_ stands for the exogenous variables; *β*_1_, *β*_2_ and *β* are vectors of parameters to be estimated; and *μ*_*i*_ is a normally distributed error term with a mean of zero and a standard deviation σ to be estimated. Here, the dependent variable *y*_*i*_ may not always be observed, and it is specially observed only when the crashes actually belong to the slight injury/KSI categories. Therefore, in the **selection model**, the dependent variable is observed if:

$$Z_{1i}\gamma_{1}+C_{i}\gamma_{2}+\upsilon_{i}=Z_{i}\gamma +\upsilon_{i}>0$$ (5)

where *Z*_*i*_ is a vector of observable features related to slight injury/KSI, which includes the overlapping variables with *X*_*i*_; *Z*_1*i*_ represents the endogenous variables that may or may not be the same as *X*_1*i*_; *γ*_1_, *γ*_2_ and *γ* are vectors of the parameters to be estimated; and *υ*_*i*_ is a distributed error term with a mean of zero and a standard deviation equal to one. This equation describes the probability that slight injury/KSI is greater than zero.

The error terms hold the following distribution:

$$\begin{array}{c} \mu_{i}\sim N(0,\sigma )\\ \upsilon_{i}\sim N(0,1)\\ corr(\mu_{i},\upsilon_{i})=\rho \end{array}$$ (6)

where *ρ* represents the correlation between the two error terms to be estimated. The parameter *λ* = *σρ*, known as the inverse Mills ratio, is the estimated selection coefficient.

There are two popular estimation methods for the model: maximum likelihood (full-information maximum likelihood, FIML) and the two-step procedure (limited-information maximum likelihood, LIML) (Leung and Yu, 1996)[33]. As the FIML estimator does not provide estimates of the “structural” variance-covariance parameters, i.e., those parameters in the unconditional distribution of the error terms, the two-step model, i.e., the LIML approach, is preferred and widely used as an alternative.

Unlike the order of the model structure, the estimation of the Heckman selection regression starts from the selection model. The estimation steps are as follows. In the first step, the **probit regression** is used to model the sample selection process in Eq 5, and then the inverse Mills ratio *λ*, the error from the probit equation explaining selection, is calculated based on the probit regression results. In the second step, the inverse Mills ratio is added to **multiple regression analysis** as an independent variable, and ordinary least square is used to provide the consistent parameter estimates in Eq 4. The likelihood function is given by

$$L=\prod_{0}\times [1-\Phi (Z_{i}\gamma )]•\prod_{1}\Phi (\frac{Z_{i}\gamma +\rho (y_{i}-X_{i}\beta )/\sigma }{\sqrt{1-\rho^{2}}})\frac{\phi ((y_{i}-X_{i}\beta )/\sigma )}{\sigma }$$ (7)

where **∏**_0_ and **∏**_1_ denote the products over the censored and uncensored samples, respectively. Φ(•) and *ϕ*(•) denote the standard normal cumulative distribution function and standard normal probability density function, respectively.

In this study, the inverse Mills ratio term includes two parts: a selection effect and an effect due to the endogeneity. If the endogeneity is absent, the endogeneity effect is zero, and the model is reduced to the general two-step selection model. The selection effect gives the expected outcome of the fully observed sample while holding the entire explanatory variables constant (including the endogenous variable), and the sign of the selection effect with the endogenous variable is determined by the correlation coefficient *ρ*. Leung and Yu (1996) [35], Mokatrin (2011) [36], Schwiebert (2015) [37] and Kaplan et al. (2016) [34] provided more detailed estimation procedures. By estimating the preceding equations, the crash rate and severity at different levels can be simultaneously and respectively calculated, and the zero samples are addressed along with the heterogeneity and endogeneity at the signalized intersections.

## Results

The results are obtained after the significant variables are examined in the model, and all of the predictor variables are verified as statistically independent without co-linearity before the model is finalized. STATA 12.0 (StataCorp LP, 2011) is used to perform the relevant analysis and estimates.

To avoid correlations between the variables, the correlation test is conducted to identify the variables to be included in the model. From the correlation matrix, the number of approach lanes, conflict points, turning movements and signal stages are highly correlated with one another. Therefore, these variables are not included in the model at the same time.

We run two models with Heckman selection regression. The first model is a selection model that determines whether there is slight injury/KSI or not. The second stage then examines the effect of the independent variables on the crash rate. Each stage has a residual for each observation, or a set of unknowns for each observation. To test for bias, we examine the relationship between the residual for the two stages (stages 1 and 2). If the unobservables in the selection model are correlated with the unobservables in the stage 2 model, we have biased estimates without correction, which implies that unobservables in the crash occurrence selection are also affecting the stage 2 model. If the unobservables in stage 1 are unrelated to the unobservables in stage 2, it indicates that stage 1 does not affect the stage 2 results. This implies that selection into the stage 2 sample is a random process, unaffected by different observables. If all of the right variables are picked for our models, and there are few unobservable variables left that affect our outcome, the chances of selection bias decrease.

The results are presented for the best model specification of slight injury and KSI as Heckman selection models in Tables 2 and 3, respectively. When *ρ* is positive, this indicates that the unobservables are positively correlated with one another; similarly, when *ρ* is negative, the unobservables are negatively correlated. As shown in Tables 2 and 3, the error terms for *ρ* are 0.778 and -0.856, meaning that the unobserved factors that cause slight injury/KSI are positively and negatively correlated with one another, respectively. The crash rate of the slight injury/KSI error terms has a large standard deviation, which implies that the heterogeneity is captured in terms of slight injury/KSI. The Wald test reveals that the joint models are preferred to the independent probit and linear regression models, with values of 45.85 and 21.90, respectively. This indicates that the two models reject the independence of the equation at the 95% confidence level.

**Table 2:** Estimated results of the Heckman selection model for slight injury.

Variables	Coefficient	Std. Err.	Z-statistic
Crash rate of slight injury			
• Reciprad	4.923*	1.005	4.90
• Cycletime	0.004*	0.001	2.63
• Tramstop	0.244*	0.112	2.19
• KLN	0.279*	0.068	4.12
• Cons	-0.714*	0.215	-3.32
Slight injury model			
• AADT	0.001*	0.001	7.43
• Reciprad	4.256*	2.094	2.03
• Tramstop	0.872*	0.240	3.63
• KLN	0.698*	0.123	5.68
• Speed	-0.230	0.004	-52.06
• Cons	10.267*	0.010	3.68
Goodness-of-fit assessment			
• Rho	0.778		
• Sigma	0.486		
• Lambda	0.378		
• Number of observations	555		
• Wald Chi-square	45.85		
• MAD	0.257		
• RMSE	0.409		

Note:

* Significant at the 5% level. $\text{The mean absolute deviation }\left(\text{MAD}\right)=\frac{1}{n}\Sigma_{i=1}^{n}\left|Y_{i}-\hat{Y_{i}}\right|$, and $\text{root mean square error}\left(\text{RMSE}\right)=\sqrt{\frac{1}{n}\sum_{i=1}^{n}{\left(Y_{i}-\hat{Y}\right)}^{2}}$, where *Y*_*i*_ is the observed value, ${\hat{Y}}_{i}$ is the predicted value and n is the number of observations.

**Table 3:** Estimated results of the Heckman selection model for KSI.

Variables	Coefficient	Std. Err.	Z-statistic
Crash rate of KSI			
• Lanewidth	-0.039*	0.014	-2.81
• Cycletime	0.007*	0.003	2.24
• KLN	0.330*	0.113	2.91
• Cons	0.854*	0.561	1.52
KSI model			
• Comveh	1.767*	0.628	2.81
• Tramstop	0.585*	0.228	2.57
• Speed	0.229*	0.003	71.98
• Cons	9.979*	0.004	2.58
Goodness-of-fit assessment			
• Rho	-0.856		
• Sigma	0.636		
• Lambda	-0.545		
• Number of observations	555		
• Wald Chi-square	21.90		
• MAD	0.385		
• RMSE	0.635		

Note:

* Significant at the 5% level.

The next thing to determine is how to interpret the estimated selection effect. To do this, we compute the average selection or truncation effect. The average truncation effect is computed as lambda*[average mills value] = 0.778*0.117 = 0.091, which depends on how much the conditional slight injury is shifted up (or down) due to the truncation effect. The interpretation of this is that injury severity with the sample set of average characteristics secured by slight injury [exp (0.091)-1*100 = 9.52% is higher than the injury severity at random from the population with the average set of characteristics. Thus, the numerical values suggest that there are positive truncation effects in these data.

### Exposure effects

#### Slight injury model

Various studies have demonstrated that AADT is significantly related to crash rate, but here AADT is shown to positively influence the slight injury, implying that traffic volume increases the possibility of slight injury. The more traffic volume on the roadway, the higher the probability that conflicts will be generated. However, because there is greater traffic volume, the vehicles may not speed much, thus the severity mostly resides in reasonable slight injury.

#### General remarks

The results show that AADT is significant in slight injury. AADT is a significant factor in crash rate, as demonstrated in various studies [9]. Nevertheless, previous studies have not verified that higher traffic volume increases the probability of slight injury, thus this study shows the potential result. Notably, this study considers both the slight injury and KSI separately, avoiding the confusing factors not addressed in the previous studies. Further research on the factors affecting slight injury and KSI severity could be helpful for the geometric designs of signalized intersections.

### Roadway characteristics

#### Slight injury model

The reciprocal of the turning radius is significantly and positively correlated with the crash rate and slight injury. A larger turning radius, i.e. smaller reciprocal of the turning radius, is accompanied by better sight distance, such that the severity of crashes decreases. However, for slight injury, the positive relation to crash rate and slight injury indicates that the possibility of crashes and slight injury still increases with the traffic flow, even under the large turning radius condition.

#### KSI model

The average lane width is negatively linked to a higher risk of KSI severity, which is uniform with previous findings from Xu et al. (2014) [9]. Currently, there is controversy about whether wider or narrower lanes are safer in roadway design, and this study suggests that wider lanes are safer, especially for KSI. Thus, it is recommended that the lanes at signalized intersections be designed wide enough to avoid KSI.

#### General remarks

The reciprocal of the turning radius is positively related to the crash rate of slight injury, implying that a small turning radius is risky for the crash rate of slight injury severity. Moreover, the reciprocal of the turning radius is related to a higher likelihood of slight injury severity, in agreement with findings from Xu et al. (2014) [9]. The findings from this study reveal the importance of designing a larger turning radius to reduce the crash rates at signalized intersections.

### Traffic characteristics

#### Slight injury model

Higher traffic speeds usually cause more severe injury and less slight injury. Traffic speed is negatively significant for slight injury, indicating that higher speeds lead to less slight injury, which supports this general point.

#### KSI model

In contrast, travel speed is positive for the KSI severity, which implies that higher travel speed leads to more KSI severity. Higher travel speed makes it difficult for the driver to maneuver the vehicle, thus the probability of running into KSI severity is larger than that associated with lower speeds.

The KSI severity at signalized intersections is positively sensitive to the proportion of commercial vehicles, in agreement with Xu et al. (2014) [9]. As the collisions with or between commercial vehicles usually have a greater force of impact and involve more people than those with or between non-commercial vehicles, a higher proportion of commercial vehicles means a higher proportion of heavy vehicles. Thus, in the event of a crash, the likelihood of a KSI is higher.

#### General remarks

The result that higher travel speeds are negatively and positively related to the probability of slight injury and KSI severity, respectively, is reasonable because higher travel speed generally causes severe conflicts. The implication is that speed limits should be posted at signalized intersections to remind the drivers to keep the travel speed steady.

The proportion of commercial vehicles is positively correlated with the probability of KSI severity, in agreement with findings from the KSI model in Xu et al. (2014) [9], but in disagreement with the findings from the slight injury model. The results suggest that the proportion of commercial vehicles should be limited so that the conflict severity can be reduced at signalized intersections. Moreover, safety education should be emphasized to reduce the aggressive behavior of commercial vehicle drivers, which is a more effective means of reducing the KSI severity for other road users than limiting the number of commercial vehicles allowed on the road.

### Signal-phasing scheme

#### Slight injury model

Cycle time is positively associated with the crash rate of slight injury. A longer cycle time is usually accompanied by longer vehicle queues and delays, implying that more vehicles arrive at the intersections during the signal cycle, and more chances conflict with each other and run into slight injury. Plus, longer cycle times may arouse some drivers’ emotions, leading to aggressive driving that ends in injuries.

#### KSI model

Cycle time is positively correlated with crash rate of KSI at signalized intersections, such that a longer cycle time increases the crash risk of KSI. For those aggressive drivers, if they know that they will have to wait for a long red light, red-light jumping may increase if they miss the last seconds of the amber light, which is a dangerous maneuver that leads to more serious crashes.

#### General remarks

Cycle time is positively linked to higher crash rates for slight injury and KSI, respectively, and this effect reflects that longer cycle times increase all types of crash rates. The results are not exactly uniform with previous findings that longer cycle times only increase the KSI crash risk [9]. The crash rate herein is investigated separately for slight injury and KSI, which may reveal the potential effect of crash rate. Longer cycle times, especially the red lights, would upset all drivers. Therefore, appropriate cycle times are not only beneficial for the signalized intersection control, but better from a safety perspective.

### Geometrical characteristics

#### Slight injury model

The presence of tram stops is positively related to the crash rate and slight injury. In Hong Kong, the tram stops are located in the center of the road, and next to the signalized intersections. More tram stops may increase the conflicts between passengers and automobiles, thus leading to more crashes. However, because the tram travels at lower speeds, and the passengers get aboard and alight at lower speeds, protected by the signals, the conflicts between passengers and the automobiles are more commonly attributed to slight injury.

#### KSI model

The presence of tram stops is positively related to the KSI severity. Similar to the Heckman model for slight injury, more tram stops may increase the conflicts between passengers and vehicles, leading to more crashes. Although the trams travel at lower speeds, some of the conflicts between passengers and the vehicles may turn into KSI if more pedestrians come across the signalized intersections.

#### General remarks

The presence of tram stops is positively related to the probability of slight injury and KSI severity, and positively significant in the crash rate for slight injury at signalized intersections. The results indicate that the more tram stops there are, the higher the likelihood of slight injury, KSI severity, and crash rate. Thus, in signalized intersections, the site selection of tram stops should be evaluated and determined according to pedestrian and traffic volume; that is, whether it is located upstream, downstream or the middle of the roadway to reduce the crash rate and severity.

### Road environment

#### Slight injury model

Compared to the traffic conditions in Hong Kong Island, the traffic in Kowloon is more complicated and worse. Thus, the road environment in Kowloon is significantly and positively related to the crash rate and slight injury. Every day, there are thousands of tourists, visitors and pedestrians in Kowloon visiting the hundreds of stores. This creates more chances for crashes, but most of them tend to be slight injury because the travel speeds are not very high due to the road environment.

#### KSI model

Similar to the Heckman model for slight injury, the traffic in Kowloon is significant and positive to the crash rate of KSI. Thousands of people walk Kowloon’s streets every day, increasing the probability of crashes, some of which may be attributed to KSI if pedestrians and the drivers are aggressive.

#### General remarks

The road environment in Kowloon is positively associated with the crash rate of slight injury and KSI, and positively related to the probability of KSI severity. The findings suggest that the road environment should be improved every now and then due to the increased probability of crashes. More attention should be paid to the sight distance of intersections, cycle times, markings and labels for pedestrians and transit stops when designing and controlling intersections.

Generally speaking, crash rate can be a predictor in the Heckman selection model for slight injury and KSI, and vice versa. In other words, the increase in the crash severity can be reflected from the decrease in crash rate since crash severity is considered as one independent variable in the crash rate function, while the increase of crash rate implies that the crash severity may be reduced, thus the endogeneity can be characterized and verified by the Heckman selection model directly.

To demonstrate the effectiveness of the proposed model, Table 4 shows the results obtained from the bivariate probit model, with which the proposed model shares some similar features. The correlation between the two severity levels (Rho) is 0.517 at the 5% significance level, different from those in Tables 2 and 3, reflecting the correlation between crash rate and injury severity. RMSE is larger in Table 4 (1.346) than in Table 2 (0.409) and 3 (0.635), and MAD value in Table 4 (0.822) shows the same trend compared with those in Table 2 (0.257) and 3 (0.385). The results in Tables 2 and 3 are apparently more specific, revealing both the crash rates for slight injury and slight injury severity and the crash rates for KSI and KSI severity simultaneously and separately. The results in Table 4 show only the slight injury and KSI severity models. Therefore, the proposed modeling approach reveals a wider coverage of performance.

**Table 4:** Estimated results of the bivariate probit model.

Variables	Coefficient	Std. Err.	Z-statistic
Slight injury model			
• AADT	0.277e-3*	0.342e-5	8.10
• Reciprad	7.242*	2.297	3.15
• Comveh	2.265*	0.622	3.64
• Tramstop	1.119*	0.253	4.43
• KLN	0.997*	0.135	7.38
• Cons	-1.995*	0.275	-7.24
KSI model			
• AADT	0.022e-3*	0.269e-5	8.19
• Reciprad	5.595*	2.178	2.73
• Comveh	3.444*	0.580	5.94
• Tramstop	0.884*	0.232	3.81
• KLN	0.497*	0.126	3.94
• Cons	-2.389*	0.257	-9.27
Goodness-of-fit assessment			
• Rho	0.517		
• Number of observations	555		
• Log likelihood at zero	-333.34		
• Log likelihood at convergence	-533.13		
• Chi-square	40.62		
• Wald Chi-square	224.37		
• MAD	0.822		
• RMSE	1.346		

Note:

* Significant at the 5% level.

## Discussion

This study uses a Heckman selection model to provide evidence about the crash rate estimation for slight injury and KSI, simultaneously and respectively, while tackling the zero samples. The two-step Heckman selection model accommodates the heterogeneity between signalized intersections and deals with the endogeneity (between crash rate and crash severity) at signalized intersections. The findings from this study reveal some directions and trends for further study and design/policies/measures.

Compared with the bivariate probit model or two-stage bivariate logistic-Tobit model used by Xu et al. (2014) [9], the Heckman selection model has the following advantages. First, its two-step procedure accommodates endogenous and heterogeneous effects by incorporating crash rate and severity into its forecast and evaluating crash rate individually according to crash severity levels. Second, by incorporating crash rate and severity, the two-step procedure reduces the effects of the overly complicated single-level modeling structure and the effects of complex modeling estimation. Third, the two-step procedure retains all of the benefits of a single-level model. Most impressive of all, the two-step procedure is easier to understand and implement than the multivariate models (Dong et al. (2014), Chiou & Fu (2015) [14, 16].

A few points about Heckman selection model are particularly worth noting. First, in Eq 5, *υ*_*i*_ is an error term or residuals of the variation in the selection model, which is a specification error or, more precisely, a case of unobserved heterogeneity determining selection bias. This specification error is considered as a true omitted-variable problem, and well taken into account when estimating the parameters of Eq 4. In other words, the impact of selection bias is neither thrown away nor assumed to be random, but is explicitly utlized and modeled in the equation estimating the outcome regression. This treatment for selection bias connotes Heckman’s contribution and distinguishes the solution to the selection bias problem from that of the traditional statisticals. Additionally, the consistent estimator of the individual parameter *ρ* (i.e. the correlation of the two error terms) and *σ* (i.e. the variance of the error term of the regression equation) were constructed to estimate the model parameters. Furthermore, the results estimated by the maximum likelihood estimator are remarkably similar to those produced using the least squares estimator. Given that the maximum likelihood estimator requires more computing time, and computing speed was considerably slower, Heckman’s least squares solution is a remarkable alternative. More important, Heckman’s solution was devised within a framework of structural equation modeling that is simple and succinct and that can be applied in conjunction with the standard framework of OLS regression. Last, the Heckman selection model depends strongly on the model being correct, much more so than ordinary regression. Running a separate probit or logit for sample inclusion followed by a regression, referred to the two-part model—not to be confused with Heckman’s two-step procedure—is an especially attractive alternative if the regression part of the model arose because of taking a logarithm of zero values. When the goal is to analyze an underlying regression model or to predict the value of the dependent variable that would be observed in the absence of selection, the Heckman model is more appropriate However, when the goal is to predict an actual response, the two-part model is usually the better choice.

Nevertheless, the dataset has its limitations. First, about 555 observations are included, and sub-crashes (zero and non-zero crashes) are very limited, thus the estimation results may be more accurate if more observations are involved. The second limitation concerns the explanatory variables. A broader range of explanatory variables could result in statistically significant coefficient estimates, thus more variables should be collected. The third limitation is that the temporal and spatial effects at signalized intersections are not addressed strongly, and the model performance may be improved by integrating the more comprehensive dataset information available for typical signalized intersections.

## Conclusions

In this paper, the crash rate and crash severity are modeled to evaluate the safety performance at signalized intersections in Hong Kong, while taking into account the heterogeneity and simultaneity of the two. The Heckman selection model, to the authors’ knowledge, is by far the first attempt in the literature on the crash rate and crash severity to simultaneously model the safety at signalized intersections. A two-step procedure is used to assess the crash rate and crash severity simultaneously and address the slight injury and KSI separately, and the zero sample issue is dealt with to accommodate the heterogeneity (i.e., shared unobserved factors) between signalized intersections and tackle the endogeneity (between crash rate and crash severity) at signalized intersections

The results of the Heckman selection model for slight injury indicate that the crash rate is positively correlated with the reciprocal of the turning radius, cycle time, the presence of tram stops and road environment in Kowloon, whereas the slight injury severity is significantly influenced by the AADT, the reciprocal of the turning radius, the presence of tram stops, the road environment in Kowloon and travel speeds. Regarding the results of the Heckman selection model for KSI, the lane width, cycle times and road environment in Kowloon increase the likelihood of crash rate for KSI. The proportion of commercial vehicles, the presence of tram stops and the travel speeds increase the likelihood of KSI severity, whereas the average lane width reduces the likelihood. The Heckman selection model also addresses the correlation between the crash rate for slight injury/KSI and slight injury/KSI severity respectively, which implies that the unobserved variables are heterogeneous between the signalized intersections in Hong Kong.

Compared with the model proposed by Xu et al. (2014) [9], the Heckman selection model involves a less complex estimation procedure. Researchers with less mathematical expertise should find it easier and more convenient to estimate the model using the associated statistical package. This may benefit practitioners and facilitate the validation process.

## Supporting information

S1 File Database (02–03)-HK-Other data. (XLS)

## References

[1] Park ES, Lord D (2007) Multivariate Poisson-lognormal models for jointly modeling crash frequency by severity. Transp Res Rec 2019: 1–6.

[2] Ye X, Pendyala RM, Washington SP, Konduri K, Oh J (2009) A simultaneous equations model of crash frequency by collision type for rural intersections. Safety Sci 47(3): 443–452.

[3] Venkataraman N, Ulfarsson GF, Shankar VN (2013) Random parameter models of interstate crash frequencies by severity, number of vehicles involved, collision and location type. Accid Anals Prev 59:309–318. 10.1016/j.aap.2013.06.021 23850546

[4] Lord D, Mannering F (2010) The statistical analysis of crash-frequency data: a review and assessment of methodological alternatives. Transp Res Part A 44(5):291–305.

[5] Kim DG., Lee Y, Washington SP, Choi K (2007) Modeling crash outcome probabilities at rural intersections: application of hierarchical binomial logistic models. Accid Anals Prev 39(1):125–134. 10.1016/j.aap.2006.06.011 16925978

[6] Kim DG, Washington SP (2006) The significance of endogeneity problems in crash models: an examination of left-turn lanes in intersection crash models. Accid Anals Prev 38(6): 1094–1100. 10.1016/j.aap.2006.04.017 16750155

[7] Ye X, Pendyala RM, Shankar V, Konduri KC (2013) A simultaneous equations model of crash frequency by severity level for freeway sections. Accid Anals Prev 57:140–149. 10.1016/j.aap.2013.03.025 23672927

[8] Ma J, Kockelman KM (2006) Bayesian multivariate Poisson regression for models injury count, by severity. Transp Res Rec 1950: 24–34.

[9] Xu X, Wong SC, Choi K (2014). A two-stage bivariate logistic-Tobit model for the safety analysis of signalized intersections. Analytic Methods in Accident Research 3–4:1–10.

[10] Abdel-Aty M, Keller J (2005) Exploring the overall and specific crash severity levels at signalized intersections. Accid Anals Prev 37(3):417–425. 10.1016/j.aap.2004.11.002 15784195

[11] Pei X, Wong SC, Sze NN (2011) A joint-probability approach to crash prediction models. Accid Anals Prev 43(3):1160–1166. 10.1016/j.aap.2010.12.026 21376914

[12] El-Basyouny K, Sayed T (2011) A full Bayes multivariate intervention model with random parameters among matched pairs for before-after safety evaluation, Accid Anals Prev 43(1):87–94. 10.1016/j.aap.2010.07.015 21094301

[13] Barua S, El-Basyouny K, Islam MT (2016) Multivariate random parameters collision count data models with spatial heterogeneity. Analytic Methods in Accident Research 9: 1–15.

[14] Dong C, Richards SH, Clarker DB, Zhou X, Ma Z (2014) Examining signalized intersection crash frequency using multivariate zero-inflated Poisson regression. Safety Sci 70: 63–69.

[15] Chiou YC, Fu C (2013) Modeling crash frequency and severity using multinomial-generalized Poisson model with error components. Accid Anals Prev 50(1):73–82. 10.1016/j.aap.2012.03.030 23200442

[16] Chiou YC, Fu C (2015) Modeling crash frequency and severity with spatiotemporal dependence. Analytic Methods in Accident Research 5:43–58.

[17] Wang C, Quddus MA, Ison SG (2011) Predicting accident frequency at their severity levels and its application in site ranking using a two-stage mixed multivariate model. Accid Anals Prev 43(6):1979–1990. 10.1016/j.aap.2011.05.016 21819826

[18] Hanley PF, Sikka N (2012). Bias caused by self-reporting distraction and its impact on crash estimates. Accid Anals Prev 49: 360–365. 10.1016/j.aap.2012.02.008 22578905

[19] Bhat CR, Born K, Sidharthan R, Bhat PC (2014) A count data model with endogenous covariates: formulation and application to roadway crash frequency at intersections. Analytic Methods in Accident Research 1: 53–71.

[20] Ding C, Ma X, Wang Y, Wang Y (2015). Exploring the influential factors in incident clearance time: disentangling causation from self-selection bias. Accid Anals Prev 85:58–65. 10.1016/j.aap.2015.08.024 26373988

[21] Miaou SP (1994) The relationship between truck accidents and geometric deisng of road sections: Poison versus negative binomial regression. Accid Anals Prev 26(4): 471–482. 10.1016/0001-4575(94)90038-8 7916855

[22] Shankar V, Milton J, Mannering FL (1997) Modeling accident frequency as zero-altered probablity processes: an empirical injury. Accid Anals Prev 29(6):829–837. 10.1016/s0001-4575(97)00052-3 9370019

[23] Carson J, Mannering FL (2001) The effects of ice warning signs on accident frequencies and severities. Accid Anals Prev 33(1): 99–109. 10.1016/s0001-4575(00)00020-8 11189127

[24] Lee J, Mannering FL (2002) Impact of roadside features on the frequency and severity of run-off-roadway accidents: an empirical analysis. Accid Anals Prev 34(2):149–161. 10.1016/s0001-4575(01)00009-4 11829285

[25] Lord D, Washington SP, Ivan JN (2005) Poisson, Poisson-gamma and zero-inflated regression models of motor vehicle crashes: balancing statistical fit and theory. Accid Anals Prev 37(1): 35–46. 10.1016/j.aap.2004.02.004 15607273

[26] Lord D, Washing SP, Ivan JN (2007) Future notes on the application of zero-inflated models in highway safety. Accid Anals Prev 29(1): 53–57. 10.1016/j.aap.2006.06.004 16949027

[27] Malyshikina N, Mannering FL (2010) Zero-state Markove switching count-data models: an empirical assessment. Accid Anals Prev 42(1):122–130. 10.1016/j.aap.2009.07.012 19887152

[28] Anastasopoulos P, Tarko AP, Mannering FL(2008) Tobit analysis of vehicle accident rates on interstate highways. Accid Anals Prev 40(2): 768–775. 10.1016/j.aap.2007.09.006 18329432

[29] Anastasopoulos P, Shankar V, Haddock J, Mannering F (2012) A multivariate Tobit analysis of highway accident-injury-severity rates. Accid Anals Prev 45(1): 110–119. 10.1016/j.aap.2011.11.006 22269492

[30] Heckman JJ (1979). Sample selection bias as a specification error. Econometrica 47:153–161.

[31] Mannering FL, Hensher DA (1987) Discrte/continuous econometric models and their application to transport analysis. Transport Reviews 7(3): 227–244.

[32] Mannering FL, Bhat CR (2014) Analytic methods in accident research: Methodological frontier and future directions. Analytic Methods in Accident Research 1: 1–22.

[33] Mannering FL, Shankar V, Bhat CR (2016) Unobserved heterogeneity and the statistical analysis of highway accident data. Analytic Methods in Accident Research 11: 1–16.

[34] Kaplan S, Nielsen TAS, Prato CG (2016) Walking, cycling and the urban form: a Heckman selection model of active travel mode and distance by young adolescents. Transp Res Part D 44: 55–65.

[35] Leung SF, Yu S (1996) On the choice between sample selection and two-part models. J. Econometrics 72: 197–229.

[36] Mokatrin L (2011) Bayesian approach sample selection bias correction in regression. Dissertation: American University, Washington, D.C.

[37] Schwiebert J (2015) Estimation and interpretation of a Heckman selection model with endogenous covariates. Empirical Economics 49(2): 675–703.

